# Expanding beyond the exome: identifying pediatric patients best suited for genome sequencing

**DOI:** 10.1007/s12687-026-00881-z

**Published:** 2026-03-30

**Authors:** Daniel Almeida do Valle, Beatriz de Haro Figueiredo, Gianna Cattoni Araldi, Giovana Pomin Barros Sachetim, Isabella Machado Barby, Lissa Ayumi Aihara, Mariana Romiti Ferreiro, Fabiana Antunes de Andrade, Mara Lúcia Schmitz Ferreira Santos, Michelle Silva Zeny, Josiane de Souza, Rare Genomes Project Consortium

**Affiliations:** 1 Hospital Pequeno Príncipe, Rua Desembargador Motta, 1070, Água Verde, Curitiba, 80250-060 Brazil; 2 Universidade Positivo, Curitiba, Brazil; 3 Hospital Israelita Albert Einstein, São Paulo, Brazil

**Keywords:** Exome Sequencing, Genome Sequencing, Pediatric

## Abstract

**Objective:**

This study aimed to compare the diagnostic yield of genome sequencing (GS) versus exome sequencing (ES) in a pediatric population, with a focus on determining whether GS offers a significant advantage over ES in identifying genetic variants.

**Methods:**

A retrospective analysis of prospectively allocated pediatric patients was performed on 297 pediatric patients who underwent next-generation sequencing. Of these, 59.6% (177) underwent only ES, 32.3% (96) underwent only GS, and 8.1% (24) had both tests. Diagnostic positivity rates were compared between ES and GS, and subgroup analyses were performed based on clinical characteristics.

**Results:**

GS demonstrated a higher positivity rate (51.7%) compared to ES (42.8%), with a 95.8% concordance between tests in patients who underwent both. However, the added diagnostic yield of GS was less than 10%. Greater GS positivity was identified in a group of children with neurological, musculoskeletal, growth disorder and craniofacial anomalies. Deep intronic variants were identified in two cases that underwent GS, but only one had also undergone ES. Limitations in medical record completeness and data quality were noted, which could affect the study’s generalizability.

**Conclusion:**

GS offered a modest increase in diagnostic yield over ES, particularly for detecting deep intronic variants. Some conditions had greater GS positivity, but the interpretation deserves caution as issues regarding homogeneity and sample selection may have influenced this result.

**Supplementary Information:**

The online version contains supplementary material available at 10.1007/s12687-026-00881-z.

## Introduction

The first widely used method for studying genetic variation was developed in the 1970s by the British chemist Frederick Sanger (Sanger et al. 1977), who received the Nobel Prize in 1980. Building upon his work, subsequent research utilized his technique, as exemplified by the Human Genome Project (1990–2003), which aimed to determine the complete sequence of the human genome (Petersen et al. 2017). Since then, human genetic variation using DNA sequencing has undergone a period of extraordinary development, both in terms of methods and in terms of increased speed and reduced costs (Conselho Nacional de Ética para as Ciências da Vida 2014; Petersen et al. 2017). As a result, current genome and exome sequencing projects have not only provided crucial information about the frequencies of variants in populations but have also shown that sequencing healthy individuals or representative population samples can lead to the discovery of relevant data about diseases (Petersen et al. 2017).

Currently, the application of NGS is essentially summarized in three: genome sequencing (GS), which encompasses all three billion base pairs); exome sequencing (ES), that covers exclusively the coding regions of the genome, which are responsible for the production of amino acids and proteins and constitute 3–4% of the entire genome; and through gene panels, previously selected, that analyses specific genes especially for the diagnosis of highly heterogeneous genetic diseases (Conselho Nacional de Ética para as Ciências da Vida 2014).

Rare diseases are defined, in the Brazilian standards, as diseases that affect up to 65 people per 100,000 individuals (Brasil 2014). Although individually rare, collectively they affect 4 to 8% of the world’s population, which translates to more than 350 million people. Notably, approximately 80% of them have a genetic etiology (Shashi et al. 2014; Bick et al. 2019). Most of these diseases affect the pediatric population, and 30% of these affected patients do not reach the age of 5 (Elliott 2020). With a traditional approach, characterized by clinical evaluation combined with selective genetic testing, 50% of children seen in genetics remain undiagnosed (Brasil 2014; Rosell et al. 2016).

Many patients, before undergoing GS and ES, go through a myriad of tests without a satisfactory answer, leading to a process known as diagnostic odyssey (Rosell et al. 2016). The diagnostic role is particularly important in pediatrics, since early identification of the disease etiology allows for more timely and specific treatments for the patient, which can improve overall prognosis and reduce complications and unnecessary interventions (Smith et al. 2019). This process can be abbreviated with the increased use of genomic testing, possibly saving economic and emotional resources of the parties involved (Shashi et al. 2014).

However, in the context of rare diseases, it is more difficult to assess the economic cost-benefit of tests, especially when comparing ES and GS, since existing treatments for rare diseases tend to be more expensive or completely absent. Nevertheless, the interruption of diagnostic costs after the test is still highlighted (Lavelle et al. 2022; Sullivan et al. 2023).

Therefore, given the growing role of NGS and its economic and social potential, it has become necessary to identify pre-test predictive factors, and which is the best test to be performed in each individual case.

## Methods

An observational cross-sectional study was conducted on 308 patients from Hospital Pequeno Príncipe (HPP), who underwent GS and/or ES sequencing between January 2021 and July 2023. This was a retrospective analysis of prospectively allocated patients. The research was approved by the Research Ethics Committee (CAAE: 75526023.0.0000.0097).

Patients were evaluated by the same medical rare disease clinical team, which performed standardized phenotypic characterization using Human Phenotype Ontology (HPO) terms. Following clinical evaluation, patients were referred for next-generation sequencing (NGS), either ES or GS. Clinical data annotated with HPO terms were forwarded to the hospital’s local laboratory, where they were registered in the laboratory information system. Random allocation to ES or GS was performed based on test availability at the time of referral and was not guided by clinical criteria, without predefined criteria related to disease severity, clinical presentation, or suspected etiology. In cases requiring urgent results and GS samples were already collected, ES was also performed to expedite analysis.

Data was collected from electronic medical records, with institutional authorization. Patients who underwent testing before 2021 or after July 2023 and those with incomplete records were excluded from the study.

ES was carried out by the Mendelics laboratory, in Brazil. Blood or buccal swab samples were collected, and DNA was extracted from the samples for genetic analyses with probes for the target regions. Next-generation sequencing was performed using Illumina technology. The Illumina fastq sequencing data were mapped to the human reference assembly, using the GRCh38 human genome as a reference. After removal of PCR duplicates (Picard) and reads without a unique mapping location, variants were extracted using Burrows-Wheeler Aligner and SAMtools and outputted by the following criteria: consensus quality > 30, SNP quality > 30 and root mean square mapping quality > 30. The potential pathogenic variants and regions with inadequate sequencing depth were confirmed using automated Sanger sequencing, which was conducted with a genetic analyzer.

GS was carried out by Rare Genomes Project Consortium. Blood samples were collected, and DNA was extracted from the samples for genetic analyses with probes for the target regions. The extracted DNA was subjected to second-generation sequencing on the Illumina platform, after mechanical fragmentation and a PCR-free protocol. The data was processed to detect point variants, copy number alterations and structural variants according to best practices for bioinformatics pipeline (Dragen DNA Germline v1.2.0). Quality plots for analysis were minimum average coverage above 20x of the bases and at least 90% depth above 15x. Variant numbering was done through the reference transcript from the base A of the ATG initiation codon aligned against the GRCh38/hg38 reference genome.

Although laboratory pipelines differed, both platforms met standard quality metrics and included CNV and mitochondrial analysis, reducing major structural differences in variant detection scope. Neither platform assessed repeat expansion disorders. Segregation was performed for phase study in biallelic variants, except in cases with the presence of biomarkers compatible with the condition studied. Segregation study was also performed for dominant variants in heterozygosity to prove De novo mutation.

The variants were described according to the nomenclature recommended by the Human Genomic Variation Society. Novel variants were classified according to the guidelines of the American College of Medical Genetics and Genomics(Richards et al. 2015) based on very low allele frequency, compound heterozygosity with a pathogenic variant, residue evolutionary conservation, and biochemical results. New variants were checked in the Human Gene Variant Database (https://www.hgmd.cf.ac.uk/) and ClinVar database (https://www.ncbi.nlm.nih.gov/clinvar/). The pathogenicity of novel missense mutations was predicted using in-silico analyses. The variants of uncertain significance were reclassified in some patients after evaluation of clinical aspects, analysis of segregation and other family members.

The negative results of GS and ES were re-evaluated by a local team to confirm the finding.

Data on participant profiles were collected, including age, sex, age at onset, comorbidities, imaging abnormalities, presence or absence of dysmorphisms, number of malformations, family history, consanguinity, diagnoses, and results of GS and ES testing. Patients who underwent both tests were included in the denominator of each respective test group when positivity rates were calculated. These data were correlated with the signs and symptoms that prompted the test, and the patients were classified based on the affected systems.

The evaluation of the data was conducted using a Microsoft Excel spreadsheet, and patients were grouped according to clinical manifestations according to Human Phenotype Ontology (Köhler et al. 2009) and summarized within the following areas: (a) growth disorders; (b) craniofacial; (c) Eye Defects; (d) Ear Defects; (e) Cutaneous; (f) cardiovascular; (g) respiratory; (h) musculoskeletal; (i) gastrointestinal; (j) genitourinary; (k) behavioral, cognitive, and developmental; (l) neurological; (m) connective tissue; (n) immunological; (o) metabolic; (p) endocrine and (q) dysmorphisms.

Data was stored and analyzed using the Statistical Package for the Social Sciences (SPSS) for Windows (version 22.0) and Microsoft Excel 2016. Descriptive statistical analyses were performed using summary measures, considering the nature of the variables involved. And inferential tests (e.g., Chi-squared, Fisher’s exact, Student’s t-test) were conducted, with statistical significance set at *p* < 0.05.

Significant differences in the positivity rate between ES and GS were adjusted using logistic regression analyses. The covariables age and clinical manifestations were included in the logistic regression model when univariate analysis presented *p* ≤ 0.2.

## Results

A total of 348 patients who underwent next-generation sequencing were primarily selected for this study. After excluding 51 patients due to incomplete medical records, 297 patients were analyzed and 8.1% (24) underwent both ES and GS, 59.6% (177) underwent ES and 32.3% (96) underwent GS.

Of the evaluated patients, 51.2% (152) were male and 48.8% (145) were female. There was no significant difference in sex distribution between the ES and GS groups (*p* = 0.299). The mean age of patients who underwent ES was 6.94 years (SD: 5.16) and for the GS group, 7.88 years (SD: 4.47).

**Fig. 1:**
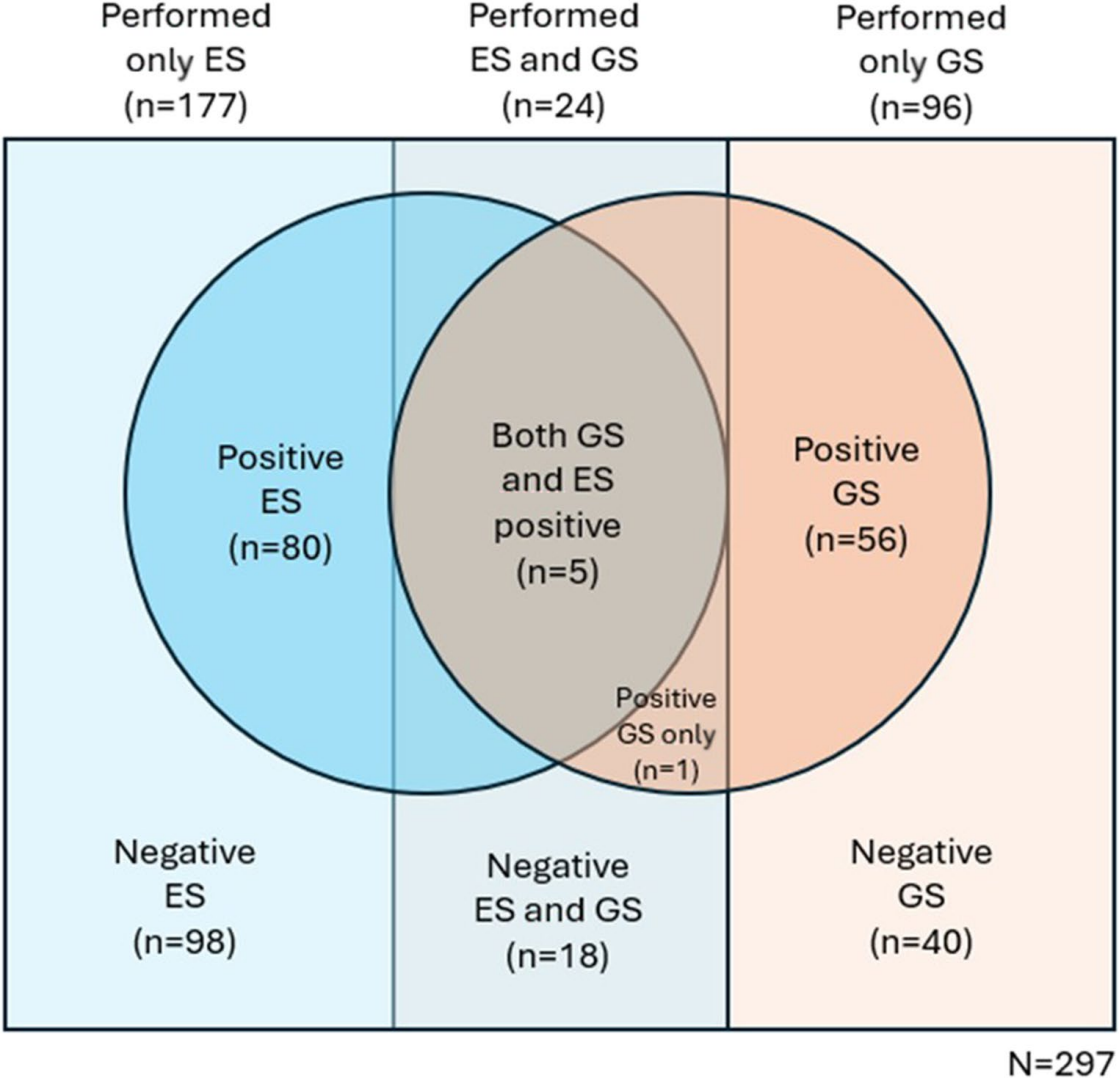
Venn diagram representing the sample distribution and positivity rate according to the test performed

In our sample, ES yielded a positivity rate of 42.2% (85) of cases, while GS showed positivity of 51.7% (62). However, among patients who underwent both exome and genome testing (24), the concordance between the two tests was 95.8%. Of these, 20.8% (5) had positive results in both tests, while 75% (18) had negative results in both. In one case, the exome test was negative, but the genome test was positive (Fig. 1). This one case was a male child with epilepsy, calcifications in the central nervous system, visual and auditory loss, periodic fever, and chronic anemia. Imaging consistent with Aicardi-Goutierres syndrome. Exome sequencing showed no abnormalities. Genome sequencing identified a de novo heterozygous variant in the IFIH1 gene: c.770-1893T > G (PM2, PM6, PP4). This deep intronic variant was not covered by exome capture design.

Initially, a total of 175 different variants were identified, including 91 pathogenic variants, 57 likely pathogenic variants, and 27 variants of uncertain significance (VUS). After individual analysis, the VUS were subsequently reclassified as likely pathogenic, primarily based on the PP1, PP4, PM3, and/or PM6 criteria. Additionally, 64 variants had not been previously described in the literature (Supplementary Table 1).

There were differences in the composition of patients assigned to ES and GS for certain conditions, including growth disorders (22.5% vs. 40.8%), craniofacial alterations (19.4% vs. 33.3%), musculoskeletal issues (30.3% vs. 42.5%), and neurological symptoms (77.8% vs. 90.8%). For other symptom groups, no significant statistical difference in patient distribution was found between ES and GS.

The positivity rates for ES and GS did not differ significantly within any clinical group where patient distribution was similar (Table 1). However, for the neurological, musculoskeletal, craniofacial alteration, and growth disorder groups, where there was an unequal distribution favoring a greater presence of patients in the GS group, the positivity rate analysis revealed statistically significant differences in these same groups (Table 1). Logistic regression was performed to adjust for differences in patient distribution between groups, allowing for a more accurate comparison of positivity rates, and the difference in positivity rates remained statistically significant in these groups. After adjustment, GS remained associated with higher positivity in neurological disorders (adjusted *p* = 0.011), Growth disorders (adjusted *p* = 0.004), Craniofacial alterations (adjusted *p* = 0.035) and Musculoskeletal disorders (adjusted p:0.021).

**Table 1:** Clinical and demographic profile of children with positive results according to the type of next generation sequencing performed

Variables		Exome (*n* = 201)	Genome (*n* = 120)	*p*	p*
Positivity rate		42.2% (85/201)	51.7% (62/120)	0.107	
Sex	Male	33.7% (36/107)	50.0% (28/56)	0.077	
	Female	52.1% (49/94)	52.1% (34/64)		
Clinical manifestations	Eye Defects	57.1% (24/42)	63.2% (24/38)	0.214	
	Ear Defects	50.0% (17/34)	42.9% (9/21)	0.512	
	Cutaneous	42.1% (8/19)	44.4% (4/9)	0.561	
	Cardiovascular	42.4% (14/33)	38.9% (7/18)	0.476	
	Gastrointestinal	39.3% (11/28)	50.0% (9/18)	0.811	
	Genitourinary	35.9% (14/39)	39.1% (9/23)	0.821	
	Behavioral, cognitive, and developmental	42.6% (55/129)	52.9% (46/87)	0.280	
	Connective tissue	52.6% (10/19)	87.5% (7/8)	1.000	
	Respiratory	27.3% (6/22)	38.9% (7/18)	0.393	
	Immunological	33.3% (4/12)	44.4% (4/9)	0.722	
	Metabolic	46.7% (14/30)	45.0% (9/20)	0.821	
	Endocrine	41.2% (7/17)	55.6% (5/9)	1.000	
	Dysmorphisms	48.4% (31/64)	53.3% (16/30)	0.211	
	Growth disorders	37.8% (17/45)	55.1% (27/49)	0.002	0.004*
	Craniofacial alterations	48.7% (19/39)	60.0% (24/40)	0.031	0.035*
	Musculoskeletal	47.5% (29/61)	64.7% (33/51)	0.017	0.021*
	Neurologic	40.4% (63/156)	53.2% (58/109)	0.002	0.011*
Family history	Consanguinity	69.2% (9/13)	50.0% (2/4)	0.120	
	Recurrence	40.0% (32/80)	45.5% (15/33)	0.371	
Brain imaging abnormalities		41.7% (48/115)	46.3% (19/41)	0.714	

*: Values are presented as unadjusted p-values; adjusted p-values (p*) were obtained using logistic regression including age and clinical manifestation as covariables

Consanguinity and recurrence did not show statistical significance (Table 1). Deep intronic variants were present in 2 cases that underwent GS. One performed ES and GS. The second deep intronic variant was identified in a patient who underwent GS only. The presence of two distinct genetic conditions in the same patient was observed in 11 patients, of these 6 were identified through GS and 5 through ES.

## Discussion

GS demonstrated a slightly higher positivity rate compared to ES, with an increase of less than 10%. These findings are consistent with those of Meienberg et al. (2016), who highlighted GS’s superior uniformity in read depth and broader genetic coverage, particularly in clinically relevant non-coding regions. GS is more effective than ES at capturing pathogenic variants in intergenic and intronic sequences and offers greater precision in identifying heterozygous positions (Lelieveld et al. 2015; Meienberg et al. 2016; Vaz-Drago et al. 2017).

Despite this slightly higher diagnostic yield, the marginal benefit of GS should be weighed against its increased cost (Alfares et al. 2018). Alfares et al. (2018) found that approximately 30% of the positive results identified by GS could have been detected through a reanalysis of ES, with GS offering only a 7% increase in detection rate compared to ES. This increase may be explained by the identification and classification of new genes during the time between the two tests or differences in interpretation and filtering processes. In this context, the length of the diagnostic odyssey directly impacts public health, as significant time and resources are spent in search of a diagnosis (Sabatini et al. 2016).

According to Meienberg et al. (2016), the decreasing cost of genetic sequencing tests is likely to promote the broader adoption of GS over ES and other genetic tests, potentially positioning GS as a future replacement (Alfares et al. 2018). Similarly, the meta-analysis by Clark et al. (2018) found no significant difference in the diagnostic utility of GS compared to ES. Given the higher cost of GS, the real benefit remains in question, especially since the statistical difference in diagnostic yield between the two tests is small. In our study, only one patient received a diagnosis via GS that was not fully identified by ES, which mirrors the findings of Alfares et al. (2018), who reported three such cases.

Previous research aligns with our findings, indicating that the diagnostic yield of genetic tests varies depending on the phenotype category (Lionel et al. 2018; Wojcik et al. 2024). Patients with neurological conditions or syndromic anomalies tend to show better outcomes (Wojcik et al. 2024). The difference between the tests was especially relevant for patients with growth disorders, craniofacial, neurological and muscular abnormalities, which is mainly due to the greater association of these groups with genetic conditions and should be taken into consideration when investigating patients with these characteristics, together with the financial and emotional costs and waiting time for delivery of the result.

The positivity rate across different clinical groups was slightly higher for GS compared to ES, although still lower than in other studies on fetal structural anomalies, which reported a 36.1% superiority for GS (Lowther et al. 2023; Wojcik et al. 2024). Additionally, GS provided a modest 0.8% increase in diagnostic yield when compared to the combined use of karyotyping, CNVs, and ES (Lowther et al. 2023). It’s important to note, however, that these studies primarily included patients with multi-systemic anomalies (50%) and very few with isolated craniofacial symptoms (less than 5%).

In this study, the use of a convenience sample poses challenges to the study’s internal validity. Although allocation was random, this was not a randomized controlled trial, and imbalance between groups occurred, and the groups were inherently heterogeneous, which can introduce biases and confounding variables. The observed differences in positivity rates between ES and GS groups could be partially influenced by the unequal distribution of clinical conditions across the groups rather than the effectiveness of the tests themselves. To address this, logistic regression analyses were performed to reduce the impact of potential biases arising from baseline inequalities by adjusting for relevant variables.

Some limitations inherent to using medical record data must be acknowledged. Patients were randomized between exome and genome, with both tests not being performed simultaneously so data quality varied significantly, with issues such as errors, incompleteness, and inconsistencies possibly affecting the reliability of our results. As ES and GS were performed in different laboratories using distinct bioinformatics pipelines, part of the observed difference in diagnostic yield may reflect laboratory-specific interpretation and reporting differences rather than intrinsic advantages of genome sequencing. Additionally, selection bias may have influenced our sample, as the available records may not accurately reflect the target population. Moreover, undocumented confounding factors could also have impacted our findings. We did not perform a formal cost-effectiveness analysis. Therefore, it is essential to consider these limitations when interpreting the findings to ensure they are assessed accurately. Future cohorts performing both tests simultaneously could allow for greater accuracy and validation of the data found in the present study.

## Conclusion

The positivity rate of genome sequencing was slightly higher than that of exome sequencing, with most variants being identified in exons, although the clinical relevance of this difference remains modest in our findings.

Children with growth disorders, craniofacial alterations, neurological symptoms, and musculoskeletal abnormalities showed a higher positivity rate for genome sequencing, though this finding should be interpreted with caution. While these groups are more likely to be associated with genetic conditions, which may explain the higher positivity rate, it is important to balance this observation with considerations such as the financial and emotional costs, as well as the extended waiting time for results. It is important to note that, despite the persistence of differences after statistical analyses and adjustments for sample distribution, the composition of the study sample may have influenced these results.

The potential contribution of deep intronic variants should also be considered when selecting the most appropriate sequencing strategy.

## Supplementary Information

Below is the link to the electronic supplementary material.

Supplementary Material 1.

## Data Availability

No datasets were generated or analysed during the current study.
